# Untargeted Volatile Profiling Identifies Key Compounds Driving the Attraction of Western Flower Thrips to *Alstroemeria* Cultivars

**DOI:** 10.3390/insects16020216

**Published:** 2025-02-16

**Authors:** Luis Gerardo Cubillos-Quijano, Marco Díaz, Ericsson Coy-Barrera, Daniel Rodríguez

**Affiliations:** 1 Biological Control Laboratory, Faculty of Basic and Applied Science, Universidad Militar Nueva Granada, Cajicá 250247, Colombia; gerardcubillos@gmail.com (L.G.C.-Q.); marco.diaz@unimilitar.edu.co (M.D.); 2 Bioorganic Chemistry Laboratory, Faculty of Basic and Applied Science, Universidad Militar Nueva Granada, Cajicá 250247, Colombia

**Keywords:** *Frankliniella* spp., HS-SPME, VOCs, feature selection, still-air olfactometry

## Abstract

Western flower thrips (WFTs) are significant pests affecting various crops globally. Developing sustainable strategies for managing WFTs is essential for improving thrips management. Ethological control methods, particularly those employing volatile organic compounds (VOCs) emitted by plants to influence insect behavior, have emerged as a promising avenue for pest management. Natural hosts such as *Alstroemeria* provide an intriguing yet underexplored opportunity for developing attractants tailored to WFT management. This study examined the behavioral preferences of WFTs towards flowers of four commercial *Alstroemeria* cultivars, focusing on variations in VOC profiles. Using headspace solid-phase microextraction (HS-SPME), VOCs were captured in vivo from cultivars with contrasting levels of WFT infestation. Gas chromatography coupled with mass spectrometry (GC-MS) was employed to analyze the VOCs, with linear retention indices aiding compound identification. An untargeted volatile profiling-based comparative analysis revealed key VOCs that differed among cultivars, shedding light on their potential correlation with WFT behavior. Behavioral assays identified three specific VOCs—butyl butyrate, 1-methylnaphthalene, and citronellyl acetate—as influential in attracting WFTs. Attraction responses were concentration-dependent, with two tested concentrations eliciting significant behavioral effects. These findings highlight the potential of these active VOCs as components of novel attractants for WFT management. The results direct future research and the development of tools to integrate ethological strategies into sustainable pest management practices for crops.

## Simple Summary

Several crops worldwide suffer damage from western flower thrips, which are small insects measuring 1.2–1.5 mm, belonging to the order Thysanoptera. To reduce reliance on chemical pesticides, scientists are exploring the use of volatile organic compounds (VOCs)—the natural scents plants emit—to attract or repel insects. This study focused on *Alstroemeria* flowers, a popular ornamental plant, to determine if their scents influence the behavior of western flower thrips, mainly if different *Alstroemeria* cultivars attract thrips in different ways because of their scents. These plant scents were collected and analyzed using multivariate statistics to determine which compounds were responsible for attracting thrips. Three specific scent compounds that seemed to be particularly appealing to thrips were identified: butyl butyrate (an aliphatic ester), 1-methylnaphtalene (an aromatic compound), and citronellyl acetate (a terpenoid). These findings are important because they provide insights into designing traps that use plant scents to attract thrips, helping farmers manage this pest sustainably, reduce chemical pesticide use, and promote healthier farming practices and environments.

## 1. Introduction

*Alstroemeria pelegrina* L. holds significant esteem in the European Union, Australia, and the United States, valued for its morphological attributes akin to the beauty of orchids. *Alstroemeria* boasts competitive advantages with captivating inflorescence beauty, vibrant colors, prolonged vase life, and a rich diversity of cultivars. Among the critical challenges to *Alstroemeria* production is the western flower thrips (WFTs) (*Frankliniella occidentalis*) (Thysanoptera: Thripidae), which is an invasive pest with global agricultural implications [1]. Western flower thrips inflict damage through feeding, oviposition, and transmission of viruses such as ANSV (*Alstroemeria* necrotic streak virus), rendering it a quarantine pest in many countries and causing critical export rejections [2,3].

Controlling WFT populations poses difficulties for farmers due to their diminutive size, cryptic habits, and distinctive morphological features, including a flattened tubular body shape and fringed wings. These characteristics facilitate their concealment in various plant parts and organs [4]. A detailed description of WFT biology and behavior, particularly in the context of pest management tactics, is provided in a recent review [5], offering additional insights into their ecological adaptations. Historically, chemical control has served as the predominant strategy; however, given its environmental and health concerns, recent research efforts have intensified toward exploring biological and ethological alternatives [5]. This shift aims to mitigate resistance and foster sustainability in managing WFTs in crops.

Over the past two decades, the significance of characterizing plant-biosynthesized volatile organic compounds (VOCs) has surged. These synthetically reproducible substances hold promise for enhancing non-biocidal integrated pest management (IPM) strategies [6]. Understanding the biology, ecology, and ethology of WFTs has facilitated the design of combined management tools that reduce harmful effects on crops [5]. For instance, the use of specialized metabolites, such as kairomones (e.g., (*S*)-(–)-verbenone, methyl isonicotinate (MIN), and *p*-anisaldehyde, among others), in color traps, has proven effective in reducing the chemical control for managing WFT populations [7,8]. Field investigations into the impact of specific compounds, such as MIN, on the capture of WFTs in nectarine crops at various phenological stages revealed a 2.4 to 3.9-fold increase in capture rates. This increase was observed despite potential competition with VOCs emitted by plants at different developmental stages [9]. Similarly, neryl (*S*)-2-methylbutanoate, the primary compound in the aggregation pheromone of two distinct *Frankliniella* species, when integrated into blue traps at medium and low doses, captured up to 1.5 times more thrips in cucumber crops than traps without the test compound [10]. Additionally, investigations into the behavioral effects of VOCs on WFTs have extended beyond the evaluation of individual attractants to include mixtures [11]. Tian et al. [12] examined a *p*-anisaldehyde and ethyl nicotinate mixture on WFT females using electroantennography tests, a Y-tube olfactometer, and greenhouse capture tests, yielding promising results for the 1:1 mixture (*w*/*w* ratio). These alternative options can potentially provide commercial benefits to relevant crops while reducing the environmental impact compared to chemical control methods.

There is a farmer-perceived notion that WFTs may prefer specific *Alstroemeria* cultivars, which can be contrary to the generalist nature of WFTs. However, specific characteristics of the plant host, particularly reproductive structures such as flowers, play a key role in their selection behavior. Factors such as the flower’s developmental stage, color, shape, and the semiochemicals it releases are known determinants [13]. Specifically, in the case of *Alstroemeria*, previous studies have demonstrated a differential attraction to various cultivars of the species, mediated by qualitative and/or quantitative VOC variations in flower emissions from commercial *Alstroemeria* cultivars, eliciting a behavioral response in WFTs and influencing host selection [14]. Therefore, this study aims to assess the behavioral response of WFTs to VOCs captured and identified from four distinct *Alstroemeria* cultivars propagated for commercial production. This aim marks the initial step in establishing the potential incorporation of these VOCs into IPM programs for WFTs.

## 2. Materials and Methods

### 2.1. Plants

Leveraging historical pest sampling data provided by growers, we selected four *Alstroemeria* cultivars with contrasting WFT occurrences during production for export, taking into account two cultivars with a high occurrence of thrips and two with a low occurrence, according to our previous findings [14]. The high-occurrence (HO) cultivars, namely ‘*Shakira*’ (yellow with lime green streaks) and ‘*Whistler*’ (white with red spots), were contrasted with the low-occurrence (LO) cultivars, ‘*Nora*’ (pink) and ‘*Himalaya*’ (white with purple spots). All cultivars, categorized as unscented (they do not generate a fragrance perceptible to humans), were procured from Könst (www.alstroemeria.com, accessed on 10 December 2024), except ‘*Nora*’, which was obtained from HilverdaKoiij (www.hilverdaflorist.com, accessed on 10 December 2024). The plants were cultivated under greenhouse conditions in Sopó, Colombia, with an average daily temperature of 13 ± 5 °C and an average relative humidity (RH) of 75 ± 15%. Flower stems at the S3–S4 opening state [15] were selected, and all plants received consistent agronomic management adhering to commercial production standards [16].

### 2.2. Insects

WFT individuals were collected using a mouth aspirator on red clover plants (*Trifolium pratense*) grown in a greenhouse at Nueva Granada Military University in Cajicá, Colombia (4°56′33.8″ N 74°00′44.4″ W). For experimental uniformity, females of unknown age and sexual status were employed to prevent potential bias related to releasing an aggregation pheromone in males [17]. The robustness and size of the abdomen facilitated the differentiation between males and females [18,19]. The thrips collected were placed in plastic boxes (25 × 17 × 11 cm, length × width × height), hermetically sealed, and equipped with appropriate ventilation at the top (6 × 15 cm, width × length). To prevent the escape of insects, a metallic mesh with a 0.03 mm aperture was securely fastened. Before the experiment, the WFT females were subjected to a 24 h starvation period within these boxes to stimulate their response to VOCs during behavioral tests. This approach assumes that hungry females are more likely to rely on VOCs to locate their food source. At the bottom of each box, an absorbent paper was positioned with moistened dental cotton pieces to ensure insect water provision. The boxes were situated in an environment maintained at a room temperature of 20 ± 1 °C, a relative humidity of 70 ± 2%, and operated under a 12:12 h photoperiod (light: dark) to mimic the conditions of Colombia and equatorial production zones.

### 2.3. In Vivo HS-SPME-Based VOC Collection from Alstroemeria Flowers

The capture of VOCs from the four commercial *Alstroemeria* cultivars was conducted in vivo using solid-phase microextraction in the headspace (HS-SPME) method [20]. The time elapsed from cutting the flower to the beginning of the collection of volatiles was approximately 30 min since preliminary investigations on *Alstroemeria* confirmed that the profiles of captured VOCs remained unaltered, whether captured from cultivated plants or 1 h harvested cut flower stems (Figure S1). To minimize the impact of environmental variations, VOC capture was conducted under controlled conditions within a climatic chamber using C720 equipment (DiEs^®^, Itagüi, Colombia). The chamber maintained a 12:12 h light:dark regime, relative humidity of 70 ± 2%, and a temperature of 20 ± 1 °C.

**Figure 1:**
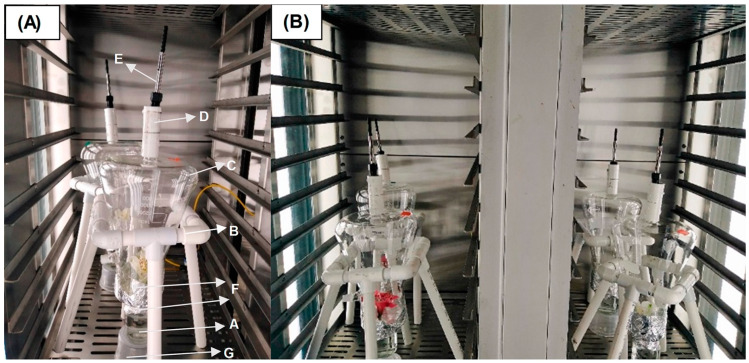
(**A**) Assembly for capturing VOCs in *Alstroemeria* flowers under controlled climatic chamber conditions. A. 113 cm^3^ bottle to keep the flower hydrated. B. Four-legged PVC support. C. Erlenmeyer flask modified and installed inverted. D. PVC support that secures the holder set–silica fiber. E. Holder with 50/30 µm DVB/CAR/PDMS fiber. F. Aluminum foil seals for the mouth of the Erlenmeyer flask and the hydration flask. G. Plastic base to support the jars. (**B**) Simultaneous assembly to capture VOCs in the four *Alstroemeria* cultivars.

All assembly materials were meticulously cleaned with neutral soap and subjected to oven-drying for four hours at 70 °C. For experimentation, a single *Alstroemeria* flower at the S3–S4 opening stage was selected, following the methodology outlined previously [21]. A four-legged support structure, constructed with polyvinyl chloride (PVC) tubes, provided stability for a 2000 mL Erlenmeyer flask modified with an opening in its base. This modification allowed the flask to be inverted and securely affixed to the support structure. Carefully introducing the flower into the inverted Erlenmeyer flask ensured no mechanical damage to its structures. The setup was sealed with aluminum foil, leaving the flower’s petiole free to immerse 4 cm into a 113 cm^3^ bottle containing water, maintaining hydration. The upper opening of the Erlenmeyer flask was sealed with Teflon tape, later intersecting with the fiber holder’s needle. This assembly allowed the needle to enter the compartment, adequately exposing the fiber and facilitating the capture of VOCs in the headspace area within the Erlenmeyer flask and around the flower. VOC capture was conducted using a 50/30 µm DVB/CAR/PDMS fiber of an SPME set (Supelco, Bellefonte, PA, USA), which was previously preconditioned (see Section 2.4), and mounted on a manual holder with a hollow stainless-steel needlepoint. The needle was secured with a cylindrical PVC support in the modified opening to introduce a previously activated silica sheet (exposed to 270 °C for 1 h) and stored at 4 °C until use (see Figure 1). The fiber was exposed to emitted VOCs for 2 h (8:00–10:00 a.m.) to avoid saturation. Following this period, the fiber was retracted and secured with the holder, refrigerated at 4 °C, and subsequently desorbed. Four assemblies were employed simultaneously, each with a flower from a different *Alstroemeria* cultivar. This design resulted in six VOC captures for each cultivar.

### 2.4. GC-MS Analysis

VOC characterization was conducted through gas chromatography coupled to mass spectrometry (GC-MS) using a Thermo Trace 1300-ISQ LT (Thermo Fisher Scientific Inc., Waltham, MA, USA). For separation, a fused silica RTX-5ms column (low polarity phase, cross-linked diphenyldimethylpolysiloxane) with dimensions of 60 m × 0.25 mm × 0.25 µm (length × internal diameter × film thickness) was employed. Before VOC collection, the DVB/CAR/PDMS coating SPME silica fiber was preconditioned at 250 °C for 30 min in the GC injection port, per the manufacturer’s guidelines. The SPME fiber was manually placed in the injection port for 2 min, with the GC inlet set at 270 °C in splitless mode. The temperature program was initiated at 40 °C for 5 min, followed by a gradual temperature increase at 6 °C/min up to 80 °C. This temperature was maintained for 5 min, followed by a further temperature increase at 4 °C/min until reaching 170 °C for 13 min, following a previous method [22]. Helium served as the carrier gas at a flow rate of 1.2 L/min. Compound identification involved a comparison of mass spectra with the NIST library (National Institute of Standards and Technology, Gaithersburg, MD, USA) (ver. 2.3, NIST/EPA/NIH 2017). This identification was complemented by linear retention index (LRI) calculations using a standard mixture of C10–C24 alkanes, analyzed under identical conditions, and the LRI values were compared with those kept in the Pherobase database (Table 1) [23]. The commercially purchased and selected VOCs (*vide infra*) were additionally analyzed using identical GC-MS conditions to confirm their presence in the studied VOC profiles (Figure S2).

### 2.5. Selected VOCs

To identify the VOCs that trigger a more significant behavioral response in thrips, three compounds were statistically selected from the most discriminating VOCs detected in the *Alstroemeria* cultivars with high WFT occurrence (following the procedure described in Section 2.7) and employed to test the behavioral response of WFTs. These compounds were commercially purchased from Sigma-Aldrich (St. Louis, MO, USA) and corresponded to butyl butyrate (standard grade, 98%), 1-methylnaphthalene (standard grade, 95%), and citronellyl acetate (standard grade, 95%). Analytical grade *n*-hexane (95%) (Merck, Darmstadt, Germany) was used as a solvent to prepare solutions and as a control for olfactometry.

### 2.6. Olfactometry Studies

**Figure 2:**
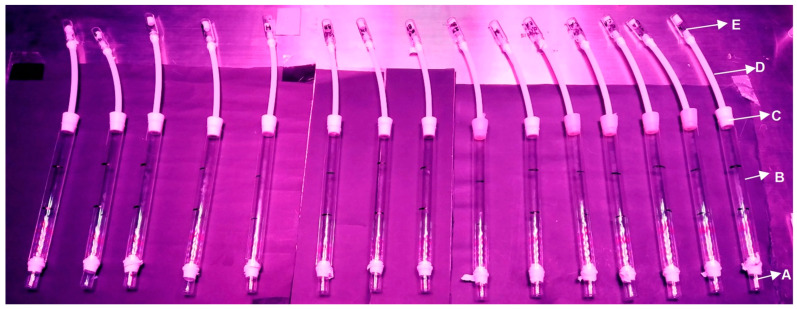
Simple tube still-air olfactometry for the three statistically selected and tested volatile organic compounds (VOCs) (i.e., butyl butyrate, 1-methylnaphthalene, and citronellyl acetate) using 15 tubes. Four concentrations of the test VOCs and the control were evaluated with *n*-hexane. A. Vial with Teflon fitting for release of thrips. B. Glass tube with three sections. C. Teflon plug with perforation. D. Teflon hose. E. Vial with Teflon fitting containing cotton for the location of VOCs.

Simple tube still-air olfactometry experiments were conducted to assess the behavioral response of female thrips to the selected VOCs. The experiments were carried out between 9 a.m. and 11:30 a.m. in a controlled environment with air conditioning set at 23 °C, complete darkness to eliminate the impact of natural light, and a full spectrum LED panel (380 nm~740 nm~45 W) (Kingbo, Boca Rato, FL, USA) with a light intensity of 1600 lux positioned on the olfactometry assembly. A custom-designed simple tube still-air olfactometer was employed to investigate the behavioral impact of the selected promising VOCs on the WFTs, focusing on the insects’ displacement within the tube. The olfactometer setup, adapted from Abtew et al. [24], consisted of cylindrical glass tubes (20 × 2 cm, length × internal diameter) connected at the top to 4 mL vials (Figure 2). Each vial contained a VOC at a specific concentration on a cotton swab for dental use. The three test VOCs were chosen based on statistical analysis and their commercial availability. The VOCs were dissolved in *n*-hexane to achieve 1%, 0.1%, 0.01%, and 0.001% *v*/*v* concentrations. Analytical-grade *n*-hexane was used as the control. The solutions and the control (2 µL each) were applied to degreased cotton pieces with an approximate volume of 0.3 cm^3^ using a pipette. A 5 min waiting period was applied to ensure complete evaporation of the *n*-hexane from the cotton pieces before proceeding with the behavioral tests.

The assembly included a Teflon hose (15 cm × 5 mm, length × internal diameter) securing the connection between the hose and the vial. A conical Teflon plug served as an adapter to connect the glass tube top, and a Swiss veil covering on the hose prevented thrips from passing from the glass tubes to the cotton pieces impregnated with the VOCs. The tube was evenly divided into upper, middle, and lower sections. For each treatment, a total of 180 females from the WFTs were evaluated. The individuals were placed in six groups of 30 females in a 4 mL vial with a Teflon O-ring to ensure a snug fit with the internal diameter of the tubes (see Figure 2). Each olfactometer was positioned on a dark surface in a completely horizontal plane (0⁰ inclination) to prevent responses induced by the negative geotropism of thrips [25]. A total of 15 simple tube still-air olfactometers were constructed to evaluate each of the three VOCs at the mentioned concentrations (twelve treatments associated with the test VOCs and three for the control treatment using *n*-hexane). The olfactometry tests involved six replicates using 30 WFT females per treatment, involving 180 WFT females (Figure 2).

The behavioral test started by opening the 4 mL vial, allowing female thrips to move through the simple tube. The response was assessed by quantifying the proportion of thrips exhibiting a movement to the upper sections of the olfactometer, and it was related to the total number of insects remaining in the bottom section. Since a low WFT presence was observed in the middle section of the olfactometer (threshold = 2 ± 1 individuals), only data from the upper and bottom sections were considered in the response, effectively transforming the experiment into a binomial one. Hence, the number of individuals in each part of the tube was recorded after 20 min, which is an appropriate time to obtain a consistent response from thrips. The upper third was categorized as “attraction,” while the lower third was labeled as “no response”. For each VOC evaluated, the proportion of individuals selecting the upper section of the olfactometer at a concentration of 0%, comprising solely hexane, served as the control.

### 2.7. Statistical Analyses

The VOC levels were determined by measuring the area under the signal for each compound detected in the total ion chromatogram of each replicate, creating a compound abundance table. These peak areas were standardized and processed according to established SPME guidelines when consistent sampling conditions across all samples were ensured [26,27]. The resulting data matrix was used to statistically select those VOCs with differential levels through univariate and multivariate analyses (a statistical procedure called feature selection [28]). One-way ANOVA was used to identify significant differences in the average relative abundance of individual VOCs between varieties, and a heatmap was employed to assess/visualize patterns between VOC normalized peak areas highlighting important VOCs among the four *Alstroemeria* cultivars. A hierarchical analysis was performed using the Ward algorithm and a heat map to classify the cultivars with high occurrence (HO) and low occurrence (LO) of thrips. A fold-change (FC) analysis (Log_2_FC values) was used to statistically determine the most discriminating VOCs in the attractive cultivars by comparing the semiquantitative levels between high-occurrence and low-occurrence pairs. The variable importance in the projection (VIP) scores, derived from partial least squares discriminant analysis (PLS-DA), was also employed for pattern recognition, focusing on *Alstroemeria* cultivars with a high WFT occurrence in the field. Finally, a receptor operating characteristic (ROC) data analysis was performed to cross-validate the contrasting levels of eight discriminating VOCs selected from the high-occurrence cultivars [29]. The statistically selected VOCs were employed to test the behavioral response of the WFT complex. The MetaboAnalyst 5.0 web tool [30] was employed for the previous statistical analyses on semiquantitative VOC-related data using default parameters.

A generalized linear model (GLM) was employed to evaluate the behavioral response of thrips concerning VOC concentrations. The comparison focused on the proportion of individuals placed in each test section (i.e., upper and lower sections). The *logit* function was used as the link function in the GLM, assuming a binomial distribution of errors. The control treatment (solvent) served as the intercept to facilitate comparisons between different concentrations of the evaluated VOCs. GLMs were performed using R software (Auckland, New Zealand) [31]. These analyses collectively provided a comprehensive understanding of the VOCs’ statistical significance and their impact on the behavioral responses of the WFT complex.

## 3. Results

### 3.1. In Vivo VOC Capture and Selection of Cultivar-Discriminating VOCs

The comprehensive examination of VOC-associated chromatographic datasets derived from the four *Alstroemeria* cultivars’ flowers revealed the presence of 53 distinct VOC-related signals during the SPME-based adsorption experiments (Figure S2). Among these, 52 VOCs were successfully identified (Table S1) and grouped into seven classes, reflecting a substantial diversity and suggesting a complex VOC profile. Terpenoids dominated the profiles, comprising 59.6% of the total relative abundance and 31 compounds, indicating their significant contribution to the VOC blend. Esters (13.5%), hydrocarbons (7.7%), and aromatic compounds (7.7%) also contributed notably, followed by alcohols (5.8%), aldehydes (3.8%), and acids (1.9%). A detected compound remained unidentified (LRI = 1742). This composition highlights the predominant role of terpenoids and esters in the chemical signature, potentially influencing ecological interactions, including plant–insect dynamics.

**Figure 3:**
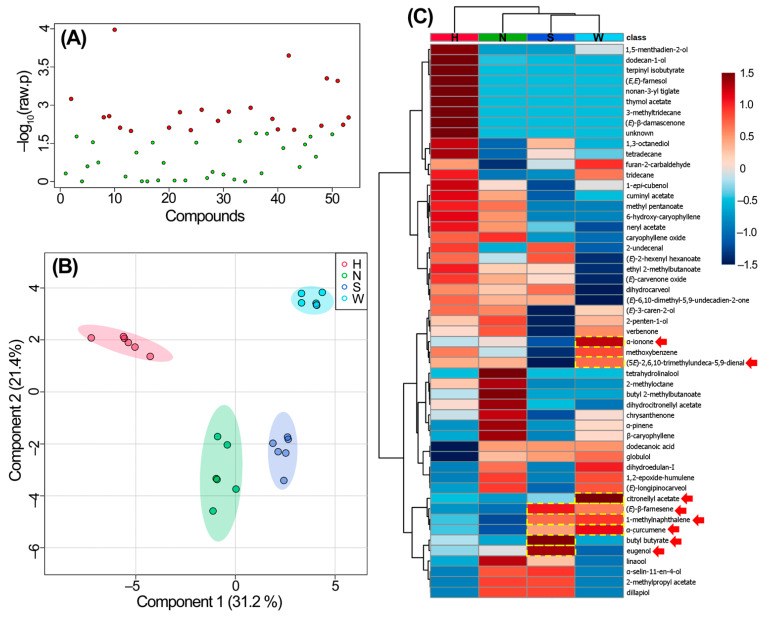
(**A**) Scatter plot from one-way ANOVA using autoscaled levels of detected volatile organic compounds (VOCs) from the four tested *Alstroemeria* cultivars. (**B**) Partial least squares discriminant analysis (PLS-DA)-derived scores plot (PC1 vs. PC2, R^2^_cum_ = 0.526) for the four tested *Alstroemeria* cultivars, H (‘*Himalaya*’), N (‘*Nora*’), S (‘*Shakira*’), and W (‘*Whistler*’). (**C**) Heatmap for the comparative analysis of the four test *Alstroemeria* cultivars. VOC names are right-sided. The color scale from 1 to −1 corresponds to variations in the autoscaled levels for each VOC per cultivar. Dual dendrograms along cultivars and VOCs were achieved through hierarchical analysis using the Ward algorithm. VOC cells highlighted in yellow and dotted squares represent the most contrasting VOCs emitted by the high-occurrence cultivars (S and W), with their names pointed out by red arrows.

The recognition of differential VOCs depending on the WFT occurrence in *Alstroemeria* cultivars requires deep comparative analysis. In this regard, using univariate and multivariate statistics within a workflow offers a comprehensive approach to identifying VOCs associated with contrasting WFT occurrences, gaining a nuanced understanding of the data. This process is known as feature selection [28]. Univariate statistics offer a focused examination of individual VOCs, while multivariate methods reveal complex relationships and patterns among these VOCs. Hence, to offer a preliminary description of the global differences in VOC levels emitted by the four cultivars, a one-way ANOVA, considering cultivar as a factor and peak area-derived VOC levels as the dependent variable, unveiled that this overall comparison exhibited VOCs (*n* = 22) with significant differences (−log_10_[*p*-value] > 1.3) (Figure 3A). This observation indicated that distinct VOC profile patterns existed among the assessed cultivars.

Subsequently, a further sPLS-DA on the VOC dataset facilitated the differentiation of cultivar-related chemical composition, illustrated by the scores plot depicting four discernible groups corresponding to each test *Alstroemeria* cultivar and their respective replicates (*n* = 6) (Figure 3B). Principal component 1 (PC1) explained 31.2% of the variance, highlighting the separation among the four cultivars, particularly distinguishing high-occurrence (HO) cultivars (‘*Whistler*’ and ‘*Shakira*’) from low-occurrence (LO) cultivars (‘*Himalaya*’ and ‘*Nora*’) in distinct plot quadrants. PC2 (21.4%) contributed to a two-subgroup splitting, with ‘*Himalaya*’ and ‘*Whistler*’ emerging as the most distant cultivars. This multivariate statistical separation demonstrated the variable chemical compositions of *Alstroemeria*-derived captured VOCs between cultivars.

A heatmap was constructed to intuitively visualize profile patterns between VOC NPAs per *Alstroemeria* cultivar (Figure 3C). In this heatmap, ‘*Himalaya*’ and ‘*Nora*’ exhibited the highest number of contrasting VOCs (21 and 13, respectively), while ‘*Whistler*’ and ‘*Shakira*’ displayed the lowest (3 and 5, respectively). A hierarchical analysis, incorporated into this heatmap, segregated Alstroemeria cultivars between HO and LO accordingly and organized VOCs from such cultivars into two primary clusters. The second cluster comprised VOCs associated with HO cultivars.

Considering the occurrence of WFTs in the field, an additional analysis was conducted to identify VOCs differentially emitted by the HO cultivars. The consistent differences intuitively visualized through the heatmap between the HO and LO cultivars, particularly focusing on VOCs with higher levels in the HO cultivars (e.g., ‘Whistler’ and ‘Shakira’), led to the preliminary selection of eight differential VOCs (highlighted in yellow, dotted squares in Figure 3C). These differential VOCs exhibited diverse chemical natures, including one ester (butyl butyrate), two aromatic compounds (1-methylnaphthalene and eugenol), and five terpenoids (citronellyl acetate, α-ionone, (E)-β-farnesene, α-curcumene, and (5E)-2,6,10-trimethylundeca-5,9-dienal). The levels of each selected VOC for each cultivar are listed in Table 1.

**Table 1:** Volatile organic compounds (VOCs) captured in vivo by HS-SPME with differential abundances in the emissions of the four *Alstroemeria* cultivars.

No.				VOC Levels ^c^			
	Volatile Organic Compounds (VOCs)			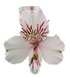	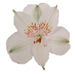	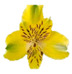	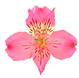
		LRI ^a^	t_R_^b^ (min)	‘Himalaya’ (LO) ^d^	‘Whistler’ (HO) ^d^	‘Shakira’ (HO) ^d^	‘Nora’ (LO) ^d^
**1**	Butyl butyrate	994	19.48	1.98 ± 0.43	n.d.	8.09 ± 2.61	n.d.
**2**	1-methylnaphthalene	1306	33.23	0.50 ± 0.13	1.25 ± 0.19	1.13 ± 0.29	n.d.
**3**	Citronellyl acetate	1349	34.77	0.60 ± 0.15	4.58 ± 1.87	0.94 ± 0.04	n.d.
**4**	Eugenol	1358	35.10	1.41 ± 0.37	n.d.	3.97 ± 1.76	1.74 ± 0.44
**5**	α-ionone	1436	37.78	0.33 ± 0.12	0.73 ± 0.09	n.d.	n.d.
**6**	(*E*)-β-farnesene	1453	38.31	0.15 ± 0.04	0.89 ± 0.26	1.12 ± 0.46	n.d.
**7**	α-curcumene	1489	39.46	0.83 ± 0.15	2.43 ± 0.28	1.74 ± 0.88	n.d.
**8**	(5*E*)-2,6,10-trimethylundeca-5,9-dienal	1500	39.82	1.31 ± 0.52	1.52 ± 0.60	n.d.	1.30 ± 0.31

^a^ LRI = linear retention indices calculated by comparison of chromatographic behavior with a series of C10-C24 *n*-alkanes. ^b^ VOCs were organized according to retention time (t_R_) on the RTX-5ms column. ^c^ VOC levels correspond to the measured peak areas in the total ion chromatogram per VOC for comparison between samples [26]. Data expressed as mean ± standard error of the mean as a result of six biological replicates; n.d. = not detected. ^d^ Occurrence of the WFT complex in the *Alstroemeria* cultivar: HO = high-occurrence; LO = low-occurrence. NPAs = normalized peak areas using *n*-tetradecyl acetate as the standard.

**Figure 4:**
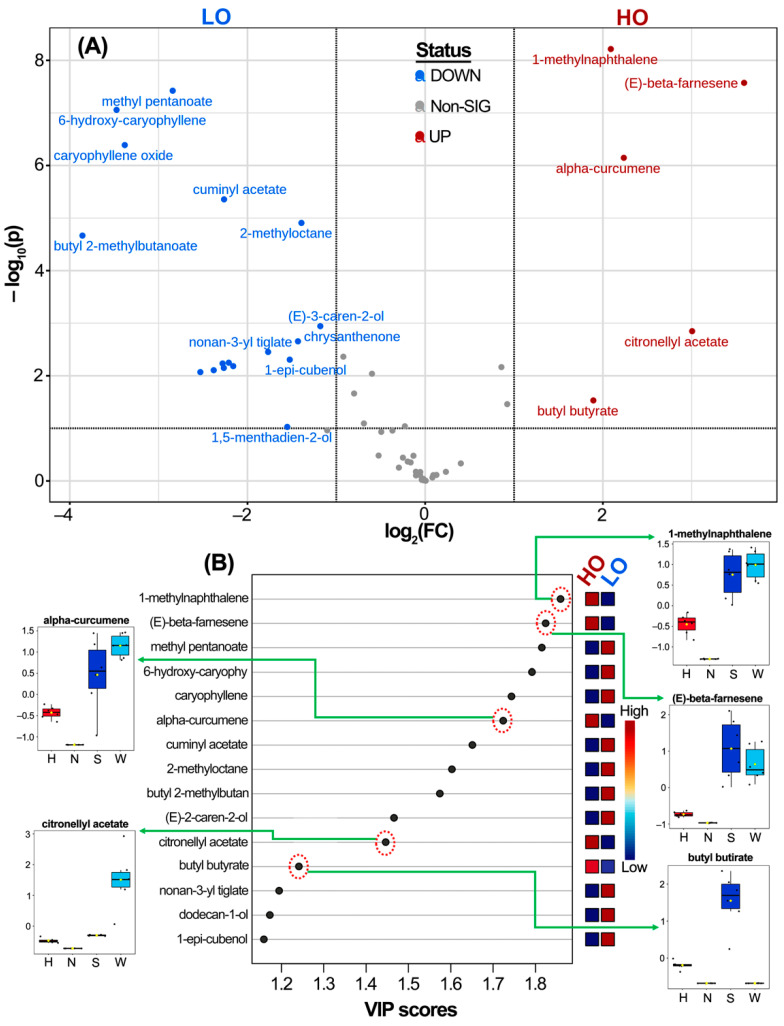
(**A**) Volcano plot comparing the autoscaled volatile organic compound (VOC) levels of WFT high-occurrence (HO, dark red) and low-occurrence (LO, blue) *Alstroemeria* cultivars; HO as an upper-class; ‘*Himalaya*’ and ‘*Nora*’ as LO cultivars; and ‘*Shakira*’ and ‘*Whistler*’ as HO cultivars. (**B**) Variable importance in the projection (VIP) plot for the fifteen top-ranked VOCs after partial least squares discriminant analysis (PLS-DA) between the VOC levels of HO and LO *Alstroemeria* cultivars. VOCs highlighted in red dotted circles represent the most differential VOCs emitted by the HO cultivars. The selected VOCs are connected to the respective boxplots that compare the VOC levels between *Alstroemeria* cultivars.

Additional supervised analysis of the VOC abundance data was conducted to enhance the selection consistency of those VOCs influencing the cultivar discrimination and WFT occurrence, segregating the dataset into HO and LO cultivars. This comparative analysis, based on the *Alstroemeria* cultivar occurrence, resulted in the statistical recognition of five VOCs with the most significant discriminating influence, determined through sPLS-DA and *t*-test/ANOVA differentiation parameters. A volcano plot was initially generated to optimize the visualization of differential VOCs for cultivars with the highest WFT complex occurrence in the field, specifically ‘*Shakira*’ and ‘*Whistler*’, employing univariate statistics. The volcano plot showed citronellyl acetate, butyl butyrate, 1-methylnaphthalene, (*E*)-β-farnesene, and α-curcumene as the most crucial, significantly differential VOCs (−log_10_[*p*-value] > 1.3, log_2_FC > 1) for discriminating HO *Alstroemeria* cultivars (Figure 4A).

This pattern recognition was extended through multivariate statistics using PLS-DA-derived variables important in the projection (VIP) scores (Figure 4B). The resulting VIP plot categorized the most important VOCs based on the VIP scores and the WFT occurrence-driven discriminating influence (red = high influence; blue = low influence). Once again, the five aforementioned VOCs were identified as critical variables for HO cultivars (VIP > 1.2, highlighted with red, dotted circles), and their boxplot-depicted distribution confirmed the statistical recognition (Figure 4B). Among these VOCs, three terpenoids (i.e., citronellyl acetate, *E*-β-farnesene, and α-curcumene), an ester (i.e., butyl butyrate), and an aromatic hydrocarbon (i.e., 1-methylnaphthalene) were top-ranked for discriminating HO-related *Alstroemeria* cultivars through the chemical composition of the captured VOCs.

An additional analysis of the area under the curve (AUC) in ROC curves was conducted to statistically validate the importance of these five VOCs in cultivar discrimination and their potential involvement in WFT complex attraction. When comparing VOC levels between HO and LO cultivars, the previously identified five VOCs were validated as differentially emitted by attractive cultivars (AUC > 0.75, *p* < 0.05, Log2FC > 1.8) (Table 2). This AUC analysis confirmed the diagnostic ability of the differential VOC variations between WFT occurrence-dependent cultivars, thereby selecting these VOC candidates for subsequent experimental verification of their chemotactic response in the WFT complex. The current study focused on performing WFT olfactometry tests for the first three selected VOCs (i.e., butyl butyrate, 1-methylnaphthalene, and citronellyl acetate). *E*-β-farnesene and α-curcumene were excluded from the present study due to challenges in sourcing these compounds from suppliers within our country. However, they are planned for inclusion in a future study.

### 3.2. Behavioral Response of WFTs to Selected VOCs

When exposed to various concentrations of the identified differential VOCs, the thrips’ behavioral response was evaluated through simple tube still-air olfactometry. We opted for this type of olfactometer configuration over other options due to its suitability for our specific objectives, as previously recommended [32]. Its advantages on WFTs include enabling continuous observation of insect behavior and minimizing issues of pseudoreplication through a straightforward setup. This olfactometer facilitated the simultaneous assessment of various VOC concentrations, replicating the calm air conditions typically found inside greenhouses. It is well suited for evaluating behavioral responses where chemotaxis, rather than anemotaxis, is the predominant mechanism involved, as is the case for WFTs [33].

**Figure 5:**
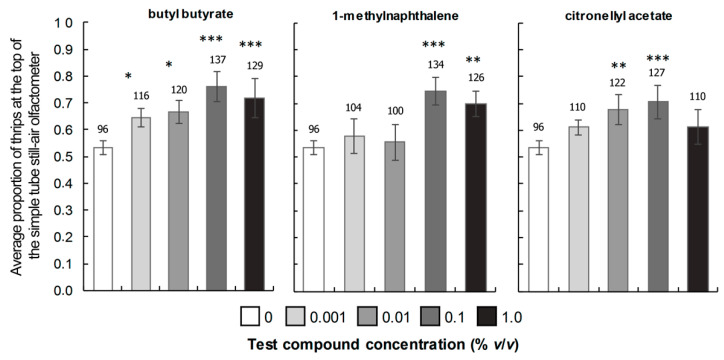
Response of WFT complex females to different concentrations of the volatile organic compounds (VOCs) identified in commercial *Alstroemeria* cultivars after 20 min. The proportion ± standard error (SE) indicates the percentage of individuals that moved toward the upper part of the simple tube still-air olfactometer (positive response). The asterisks indicate the degree of significance when compared with the concentration 0 (*n*-hexane) according to the values of *p* < 0.001 (***), *p* < 0.01 (**), and *p* < 0.05 (*) in the GLM. The numbers above the bars represent the total number of females that showed a positive response to each concentration and tested VOC among the total number of individuals (*n* = 180), which was subdivided into six groups of 30 females each.

As shown in Figure 5, the three tested compounds attracted WFTs at several concentrations, with a noticeable dose–response relationship that was particularly evident for the ester butyl butyrate. In this case, the proportion of attracted individuals was statistically higher than the control (<*n*-hexane) for all concentrations, demonstrating highly significant differences for the highest concentrations [i.e., 0.1% (*p* < 0.001) and 1.0% (*p* < 0.001)]. In contrast, significant differences were observed for the lowest concentrations [i.e., 0.01% (*p* < 0.05) and 0.001% (*p* < 0.05)]. The proportion of females moved toward the upper part at 0.1%, and 1% were 76% and 72%, respectively, while for the other two concentrations (0.01% and 0.001%), it was 67% and 64%. In the case of 1-methylnaphthalene, the proportion of attracted individuals was highly significant compared to the control at 0.1% and 1% (*p* < 0.01). For these two concentrations, the proportion of individuals was 74% and 70%, respectively. Regarding citronellyl acetate, two concentrations exhibited a proportion of attracted individuals statistically higher than the control, evidencing highly significant differences at 0.1% (*p* < 0.001) and 0.01% (*p* < 0.001). The proportions of individuals for these concentrations were 71% and 68%, respectively (Figure 5).

## 4. Discussion

Despite their shared genus and identical agronomic management, this study effectively demonstrated variations in the captured VOC profiles among four Alstroemeria cultivars. The ‘*Himalaya*’ cultivar exhibited the highest VOC richness compared to the other three cultivars despite low thrips occurrence in the field. In contrast, the ‘*Shakira*’ cultivar showed a higher abundance of eugenol, which may explain its increased thrips occurrence, as eugenol is a known attractant for WFTs [8]. Butyl butyrate emerged as the VOC most significantly distinguishing the ‘*Shakira*’ cultivar due to its elevated abundance. Conversely, citronellyl acetate and 1-methylnaphthalene were more abundant in the ‘*Whistler*’ cultivar, which also showed higher thrips occurrence. The ‘*Nora*’ cultivar had a moderate number of VOCs and was not attractive to thrips, aligning with farmer observations. Although its captured VOC profiles included eugenol [8] and (5*E*)-2,6,10-trimethylundeca-5,9-dienal—a compound reported as a termite sex pheromone [34]—the abundance of these compounds appeared too low to elicit an attractant effect.

In addition, this exploration exclusively focused on flower-emitted VOCs, excluding leaves and other plant structures. By eliminating green leaf volatiles (GLVs) from the analysis, we avoided confounding effects from other plant organs that might simultaneously attract WFTs. Research by Nyasani et al. [35] highlighted how host vegetative states can alter thrips’ feeding and oviposition behaviors, while Yang et al. [36] demonstrated that WFTs select hosts based on specific VOC levels, such as linalool emitted by both flowers and leaves. These facts may explain the varying frequencies of WFTs among *Alstroemeria* cultivars under identical growing conditions.

Terpenoids are predominant contributors to VOC emissions in ornamental flowers, including *Alstroemeria*, rose, and *chrysanthemum* [20,37,38]. In the present study, terpenoids accounted for 59.6% of the identified VOCs (*n* = 53), with 60% of the most contrasting VOCs among cultivars belonging to this group. Terpenoids play a crucial ecological role in interspecific plant communication, mediating responses to environmental stress and herbivory [39,40]. Abiotic factors (e.g., light and temperature) and biotic factors (e.g., herbivory) may influence the VOC composition of *Alstroemeria* cultivars. Kigathi et al. observed differences in VOC emissions from *T. pratense* under field and laboratory conditions, particularly when herbivory occurred or under variations in light and temperature [41]. Herbivory-induced VOC variations among *Alstroemeria* cultivars may strengthen metabolic defenses and differentially affect thrips occurrence. Moreover, plants’ phytosanitary and nutritional status can induce shifts in the preferences of pest insects. For instance, Mwando et al. [42] observed increased methyl salicylate emissions in virus-infected corn plants, which attracted vector species, while Karlsson et al. [43] reported that potato tuber moths (*Tecia solanivora* (Lepidoptera: Gelechiidae)) distinguish VOC emissions from healthy versus diseased tubers, selecting healthy ones for oviposition to ensure optimal conditions for offspring development.

Variability in VOC profiles among cultivars has been previously documented. Aros et al. [37] noted variations in VOC emissions among four *Alstroemeria* hybrids, while Avellaneda et al. [20] reported similar findings in commercial rose cultivars. These differences may stem from genetic differences in the parental lines used to develop these cultivars. The crosses involved native alstroemerias from diverse geographical regions, including Peru, Chile, and Brazil. The origin of cultivars plays a pivotal role in determining the genetic expression of each plant [16]. It dictates whether Alstroemerias exhibit specific aromas or not, as certain genes encode enzymes influencing aroma regulation and production in *Alstroemeria* flowers [37]. The inheritance of these traits is closely tied to genetic diversity and specific regulatory factors.

All the evaluated *Alstroemeria* cultivars were designated as unscented based on information provided by the breeders. However, this classification does not imply that the flowers cease to produce VOCs or that this ecological mechanism in their natural environment is stopped. The observed lack of aroma in alstroemerias might be attributed to genetic enhancements to improve specific traits [44]. This decrease or imperceptibility of aroma to humans could result from a potential negative correlation between fragrance and vase life, with the latter being a highly desirable trait in commercial cultivars [45,46]. Additionally, the emission of allergenic compounds in some alstroemeria cultivars, leading to mutagenesis procedures to limit their production, might have inadvertently contributed to a selection for reduced fragrance production [47]. It is important to note that the abundance and concentration of VOCs in *Alstroemeria* are not directly correlated with human perception, aligning with studies by Aros et al. [48] in *Lilium* spp., *Freesia* sp., and *Chrysanthemum* spp. flowers. This fact concurs with the understanding that, in nature, VOC production serves as a biological strategy for flowering plants to enhance reproduction and propagation, differing from the agronomic or commercial goals of cultivation. As the flower is the reproductive organ of higher plants, the emission of VOCs during flowering possibly constitutes a strategy to ensure pollination and fertilization, often mediated by terpenoids [49]. Each *Alstroemeria* cultivar has been meticulously developed through genetic improvement to enhance various agronomic characteristics, such as flower size, cold or heat tolerance, and stem quality, among others [50]. However, this improvement could potentially impact the biological characteristics and mechanisms of the flowers under natural conditions, including VOC emission, to ensure efficient reproduction.

During olfactometry tests, WFT females were significantly attracted to various concentrations of the three flower VOCs, which were statistically ranked as the top choices. This observation agrees with Nyasani et al. [35], who demonstrated a preference by WFT females for flowering plants as sites for oviposition. The three selected VOCs—i.e., butyl butyrate, 1-methylnaphthalene, and citronellyl acetate (Table 2)—lack previous reports as behavior mediators for Thysanoptera:Thripidae insects. Notably, butyl butyrate displayed differences from the control across all assessed concentrations, with attraction percentages exceeding 70% in two concentrations (i.e., 0.1% and 1%). Previous studies have implicated this compound in chemical communication as an allomone (e.g., *Elmaspoda valga*), pheromone (e.g., *Alydus pilosulus*), kairomone (e.g., *Rhagoletis pomonella*), and as an attractant (e.g., *Psyttallia concolor*) [23]. On the other hand, 1-methylnaphthalene has been reported as an allomone (e.g., *Aedes aegypti*) [23], while citronellyl acetate has been identified as an allomone (e.g., *Pithitis smaragdula*) and pheromone (e.g., *Vespa crabo*) [23], and as a component of *Citrus* plants with acaricidal effect [51]. This study constitutes the first report on the effects of these VOCs on the WFT complex and the detection of these VOCs in *Alstroemeria* plants.

This study employed a simple tube still-air olfactometer [24,52]. This device does not allow the evaluation of anemotactic responses, which are common in many insect species. However, wind currents are uncommon under greenhouse conditions, and the environment is typically characterized by relatively calm air. To ensure consistency in air conditions during olfactometry tests, we opted to conduct evaluations using a system based on static air rather than dynamic air, such as Y-olfactometry. It is assumed that under still air conditions, like those found in greenhouses, the response of thrips primarily involves chemotaxis rather than anemotaxis. Thrips are known to be weak fliers, relying more on drag than lift during flight activity. Therefore, it is unlikely that host-finding in thrips involves anemotaxis, with chemotaxis under calm air conditions serving as the primary mechanism in the host-finding process [33,53].

These findings open new avenues for research into promising VOCs that modulate chemotactic responses in the WFT complex. Furthermore, these results enhance our understanding of the influence of these VOCs on the behavior of these insects, facilitating the design of more selective and effective management strategies. The laboratory tests conducted in this study represent a crucial step in recommending these VOCs for field management strategies. In this context, future research must focus on evaluating the effectiveness of these VOCs in greenhouse conditions. If their activity is confirmed under such conditions, the following steps would involve developing and formulating a product for use in crop management. On the other hand, although this study did not assess the impact of VOC mixtures emitted by *Alstroemeria* flowers, the behavioral tests with individual attracting VOCs provide valuable insights into the WFT response to statistically selected compounds from attracting *Alstroemeria* cultivars. Consequently, future studies are recommended to evaluate mixtures at defined ratios to investigate potential synergistic effects that could amplify attraction, ultimately aiding the development of more effective ethological control strategies.

## 5. Concluding Remarks

The current study successfully demonstrated distinctive VOC profiles among four *Alstroemeria* cultivars with contrasting WFT occurrence, leading to the discovery of a positive chemotactic response of WFTs by three specific *Alstroemeria*-derived VOCs with varying levels between attractive and non-attractive cultivars. This fact suggests a potential genetic basis for the observed differences in VOCs, even among cultivars sharing similar agronomic management practices. The identified differential VOCs and variations in WFT occurrence were associated with a chemotactic response, supported by the behavioral response assessed for the statistically selected VOCs in simple tube still-air olfactometry tests. Notably, butyl butyrate elicited significant attracting responses across all tested concentrations (0.001%, 0.01%, 0.1%, and 1%), while 1-methylnaphthalene and citronellyl acetate triggered WFT responses at two concentrations each (0.1% and 1%, and 0.01% and 0.1%, respectively). These findings hold promise for developing innovative products to manage WFT populations in *Alstroemeria* flower crops within non-chemical IPM programs.

## Figures and Tables

**Table 2:** Results of the AUC analysis of the ROC curves for VOCs with contrasting levels between commercial *Alstroemeria* cultivars.

t_R_^a^	Contrasting VOCs ^b^	AUC ^c^	*p*-Value ^d^	Log_2_FC ^e^
19.48	Butyl butyrate	0.75	2.94 × 10^−2^	1.893
33.23	1-methylnaphthalene	1.00	6.09 × 10^−9^	2.090
34.77	Citronellyl acetate	1.00	1.42 × 10^−3^	3.005
38.31	*E*-β-farnesene	1.00	2.68 × 10^−8^	3.589
39.46	α-curcumene	0.95	7.14 × 10^−7^	2.236

^a^ Retention time (t_R_). ^b^ Contrasting volatile organic compounds (VOCs) after dual comparison between WFT high-occurrence (HO) *Alstroemeria* cultivars (‘*Shakira*’ and ‘*Whistler*’) and low-occurrence (LO) *Alstroemeria* cultivars (‘*Himalaya*’ and ‘*Nora*’). ^c^ Area under the curve (AUC) from receiver operating characteristic (ROC) curves. ^d^ *p*-value from unpaired *t*-test. ^e^ Log_2_FC = binary logarithm of fold change (FC).

## Data Availability

The original contributions presented in this study are included in the article; further inquiries can be directed at the corresponding authors.

## Supplementary Materials

The following supporting information can be downloaded at https://www.mdpi.com/article/10.3390/insects16020216/s1: Table S1: Volatile organic compounds (VOCs) captured in vivo by HS-SPME from four *Alstroemeria* cultivars; Figure S1: Total ion chromatograms for comparing the VOCs captured between (A) 1 h harvested cut flower stems and (B) uncut flower stems of an Alstroemeria cultivar (‘Whistler’); Figure S2: Total ion chromatograms (TICs) of selected replicates of those VOC profiles captured from 1 h harvested cut flowers of four *Alstroemeria* cultivars: (A) ‘Himalaya’; (B) ‘Whistler’; (C) ‘Shakira’; and (D) ‘Nora’. (E) TICs for the three-component mixture of three standards selected for behavioral analysis.
