# Supplementary material for: Untargeted Volatile Profiling Identifies Key Compounds Driving the Attraction of Western Flower Thrips to Alstroemeria Cultivars
Source: Insects. 2025 Feb 16;16(2):216. doi: 10.3390/insects16020216 (PMC11857083; doi:10.3390/insects16020216)
Supplement: Supplementary file 1 [file insects-16-00216-s001.zip › insects-3414071-supplementary.pdf]

Supplementary Material

Untargeted Volatile Profiling Identifies Key Compounds Driving the Attraction of Western Flower Thrips to *Alstroemeria* Cultivars

Luis Gerardo Cubillos-Quijano [1], Marco Díaz [1], Ericsson Coy-Barrera [2,*] and Daniel Rodríguez [1,*]

Content

	Page
**Table S1.** Volatile organic compounds (VOCs) captured in vivo by HS-SPME from four Alstroemeria cultivars	2
**Figure S1.** Total ion chromatograms for comparing the VOCs captured between **(A)** 1-h-harvested cut flower stem and **(B)** uncut flower stem of an Alstroemeria cultivar ('Whistler').	3
**Figure S2.** Total ion chromatograms (TIC) of selected replicates of those VOC profiles captured from 1-h-harvested cut flowers of four *Alstroemeria* cultivars **(A)** 'Himalaya'; **(B)** 'Whistler'; **(C)** 'Shakira', **(D)** 'Nora'. **(E)** TIC for the three-component mixture of three standards selected for behavioral analysis.	3

Table S1. Volatile organic compounds (VOCs) captured in vivo by HS-SPME from four *Alstroemeria* cultivars (**H** = 'Himalaya'; **W** = 'Whistler'; **S** = 'Shakira'; **N** = 'Nora')

#	Name of identified VOC [a]	RT [b] (min)	LRI [c] (exp)	LRI [d] (rep)	*Alstroemeria* cultivars			
					H	W	S	N
**1**	2-penten-1-ol	11.29	772	767	7.67±3.47	7.82±0.45	4.86±1.26	8.7±4.57
**2**	2-methylpropyl acetate	11.86	786	780	0±0	0±0	0.35±0.09	0.36±0.05
**3**	methyl pentanoate	13.3	822	825	1.14±0.52	0±0	0±0	0.95±0.18
**4**	furan-2-carbaldehyde	13.68	831	830	2.19±1.93	2.26±0.8	2.1±0.85	1.9±0.88
**5**	ethyl 2-methylbutanoate	14.31	847	846	1.83±0.9	0.51±0.11	1.33±0.48	1.38±0.45
**6**	2-methyloctane	14.85	860	861	6.35±2	5.7±1.18	2.94±1.9	9.78±5.88
**7**	methoxybenzene	17.02	918	917	2.44±1.28	2.7±0.51	0±0	1.52±1.68
**8**	alpha-pinene	17.92	946	950	0±0	0.22±0.08	0±0	0.5±0.17
**9**	butyl butyrate	19.48	994	994	1.98±0.4	0±0	8.09±2.61	0±0
**10**	butyl 2-methylbutanoate	21.81	1043	1043	0.3±0.05	0±0	0±0	1.09±0.07
**11**	tetrahydrolinalool	24.33	1093	1097	0±0	0±0	0±0	0.38±0.14
**12**	linalool	24.98	1107	1105	1.06±0.23	2.48±0.7	1.78±0.75	1.53±0.65
**13**	chrysanthenone	25.97	1129	1125	0.57±0.2	0.65±0.42	0±0	1.26±0.1
**14**	1,5-menthadien-2-ol	28.41	1185	1183	0.78±0.51	0.24±0.05	0±0	0±0
**15**	dihydrocarveol	28.82	1194	1195	0.9±0.35	0.77±0.25	1.37±0.98	0.88±0.47
**16**	verbenone	29.42	1208	1204	0.92±0.38	1.24±0.53	0.82±0.45	0.95±0.3
**17**	(*E*)-3-caren-2-ol	30.36	1232	1227	2.74±0.69	2.16±0.23	0±0	2.6±0.76
**18**	(*E*)-carvenone oxide	31.84	1270	1272	0.64±0.12	1.56±0.79	1.93±0.97	1.01±0.36
**19**	1,3-octanediol	31.97	1274	1275	0.74±0.04	0.55±0.03	0.64±0.06	0.5±0.24
**20**	dihydroedulan-I	32.79	1295	1292	0±0	0.5±0.18	0±0	0.41±0.12
**21**	tridecane	33.01	1300	1300	4.15±1.12	4.64±0.8	3.99±1.32	3.92±1.13
**22**	1-methylnaphthalene	33.23	1306	1312	0.5±0.13	1.25±0.19	1.13±0.29	0±0
**23**	dihydrocitronellyl acetate	33.81	1322	1320	0.63±0.15	0.59±0.2	0.61±0.09	0.69±0.13
**24**	citronellyl acetate	34.77	1349	1354	0.6±0.15	4.58±1.87	0.94±0.04	0±0
**25**	eugenol	35.1	1358	1356	1.41±0.37	0±0	3.97±1.76	1.74±0.38
**26**	thymol acetate	35.2	1361	1362	2.23±0.62	0±0	0±0	0±0
**27**	neryl acetate	35.29	1363	1365	1.28±0.09	1.01±0.36	1.56±0.56	1.21±0.35
**28**	2-undecenal	35.56	1371	1368	1.89±0.42	1.12±0.8	1.86±0.32	1.31±0.5
**29**	3-methyltridecane	35.69	1374	1372	5.5±1.88	0±0	0±0	0±0
**30**	(*E*)-2-hexenyl hexanoate	35.71	1375	1375	4.28±0.73	2.74±1.26	4.34±1.8	3.65±1.01
**31**	(*E*)-beta-damascenone	36.21	1388	1391	1.29±0.38	0±0	0±0	0±0
**32**	tetradecane	36.55	1398	1400	5.72±2.82	4.05±1.55	4.42±4.31	3.4±1.9
**33**	cuminyl acetate	37.29	1421	1419	1.58±0.7	0.48±0.07	0±0	1.09±0.35
**34**	β-caryophyllene	37.57	1429	1428	3.43±0.69	3.48±0.28	3.42±1.96	3.56±0.37
**35**	α-ionone	37.78	1430	1426	0.33±0.12	0.65±0.07	0±0	0.38±0.1
**36**	(*E*)-β-farnesene	38.31	1456	1458	0.15±0.04	0.89±0.26	1.12±0.46	0±0
**37**	(*E*)-6,10-dimethyl-5,9-undecadien-2-one	38.52	1457	1455	3.81±1.34	1.66±0.25	3.49±1.49	3.45±2.28
**38**	terpinyl isobutyrate	38.89	1471	1471	1.29±0.12	0.64±0.2	0.64±0.19	0.65±0.12
**39**	dodecan-1-ol	39.07	1476	1473	1.71±0.29	0.62±0.15	0.63±0.16	0.66±0.15
**40**	α-curcumene	39.46	1489	1487	0.83±0.15	2.43±0.28	1.74±0.88	0±0
**41**	(5E)-2,6,10-trimethylundeca-5,9-dienal	39.82	1502	1501	1.31±0.52	1.52±0.6	0±0	1.3±0.35
**42**	nonan-3-yl tiglate	39.97	1503	1506	0.87±0.1	0±0	0±0	0±0
**43**	dodecanoic acid	42.44	1556	1559	0±0	0.91±0.04	0.9±0.24	0.89±0.26
**44**	globulol	43.29	1574	1576	0.69±0.16	4.37±2.46	3.98±2.33	3.71±1.45
**45**	caryophyllene oxide	43.3	1581	1581	3.44±1.32	0±0	0.6±0.09	3.81±2.26
**46**	1,2-epoxide-humulene	44.46	1606	1607	0±0	2.88±1.59	0±0	2.96±0.49
**47**	1-*epi*-cubenol	44.91	1616	1616	5.05±3.04	2.54±0.97	0±0	2.71±0.14
**48**	dillapiol	45.19	1622	1622	0±0	0±0	1.01±0.35	1±0.29
**49**	(*E*)-longipinocarveol	45.63	1634	1634	0.43±0.12	2.03±0.28	0±0	2.14±0.34
**50**	6-hydroxy-caryophyllene	46.24	1643	1643	1.82±0.76	0±0	0±0	1.29±0.26
**51**	α-selin-11-en-4-ol	46.72	1655	1655	0±0	0±0	11.22±1.56	10.51±1.77
**52**	(*E*,*E*)-farnesol	49.48	1723	1722	0.64±0.24	0±0	0±0	0±0
**53**	unknown	50.21	1742	-	1.08±0.34	0±0	0±0	0±0

[a] Identified VOC according diagnostic analysis; [b] RT = Retention time; [c] LRI (exp) = Linear retention index calculated from experimental GC-MS analysis; [d] LRI (rep) = Reported linear retention index in pherobase.

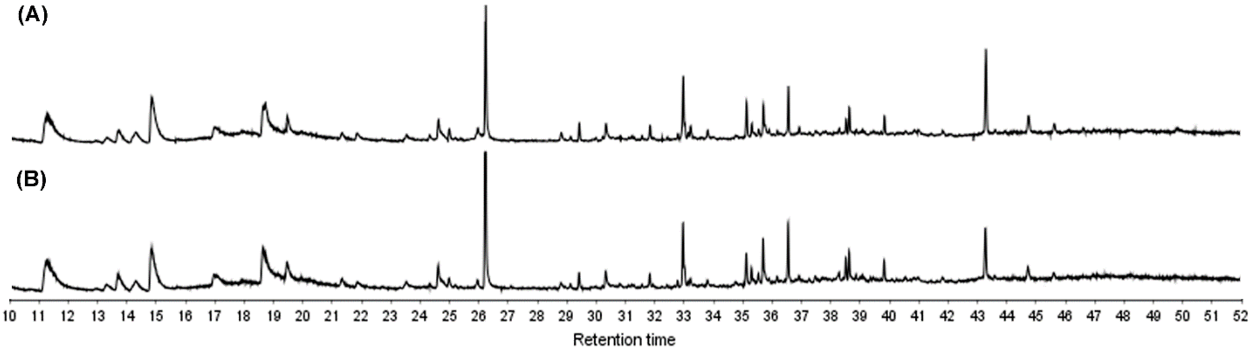

Figure S1. Total ion chromatograms for comparing the VOCs captured between **(A)** 1-h-harvested cut flower stem and **(B)** uncut flower stem of an Alstroemeria cultivar ('Whistler').

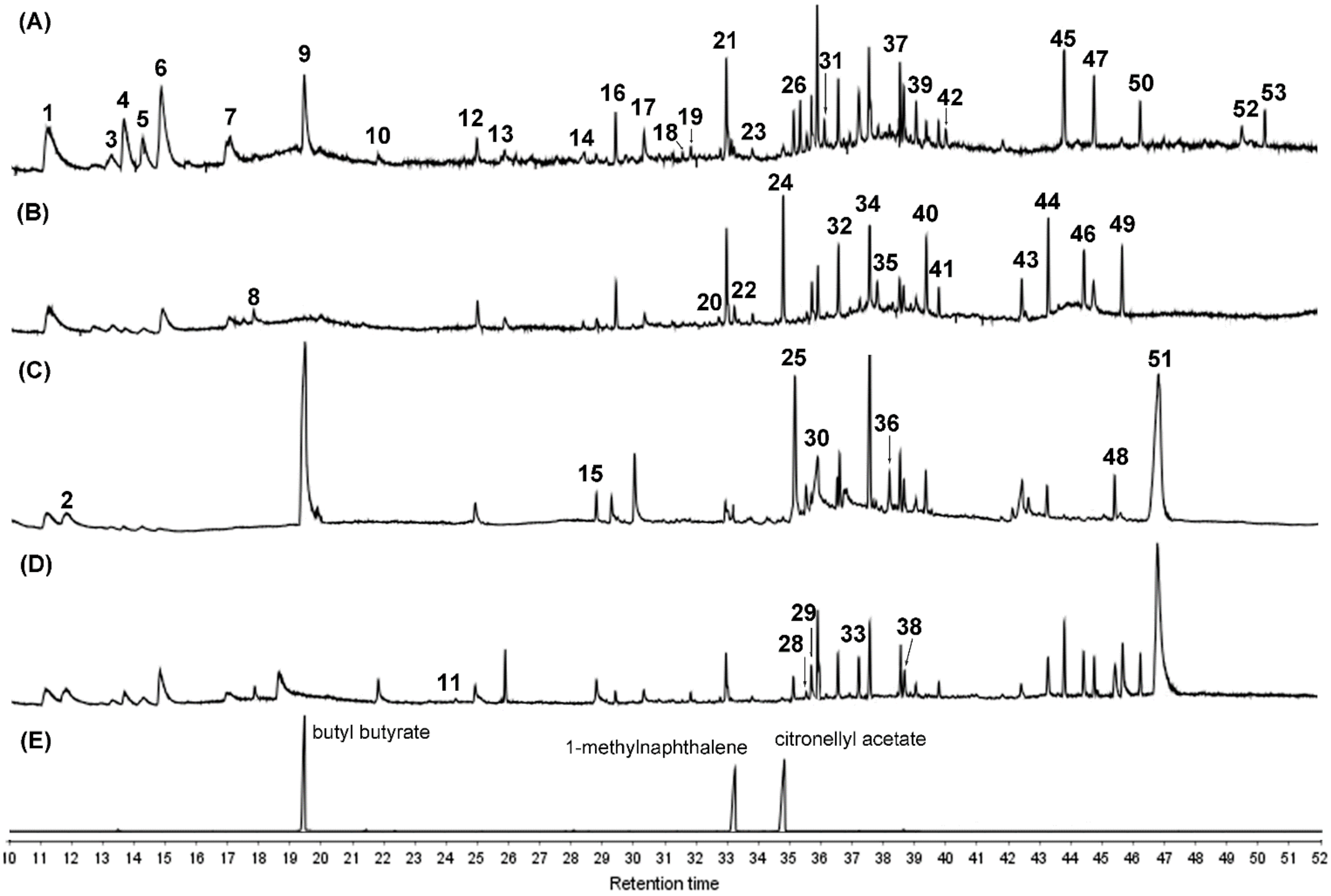

Figure S2. Total ion chromatograms (TIC) of selected replicates of those VOC profiles captured from 1-h-harvested cut flowers of four *Alstroemeria* cultivars **(A)** 'Himalaya'; **(B)** 'Whistler'; **(C)** 'Shakira', **(D)** 'Nora'. **(E)** TIC for the three-component mixture of three standards selected for behavioral analysis. Bold numbers over signals corresponding to identified VOCs listed in Table S1 for the four *Alstroemeria* cultivars.

.
